# Aqueous humour concentrations of PEDF and Erythropoietin are not influenced by subthreshold micropulse laser treatment of diabetic macular edema

**DOI:** 10.1042/BSR20190328

**Published:** 2019-06-18

**Authors:** Edoardo Midena, Silvia Bini, Luisa Frizziero, Elisabetta Pilotto, Graziana Esposito, Alessandra Micera

**Affiliations:** 1 Department of Ophthalmology, University of Padova, Padova, Italy; 2 IRCCS – Fondazione Bietti, Rome, Italy

**Keywords:** diabetes, diabetic macular edema, diabetic retinopathy, subthreshold micropulse laser

## Abstract

**Purpose:** To determine if aqueous humour (AH) concentrations of Retinal Pigment Epithelium (RPE)’s biomarkers are modified after subthreshold micropulse laser (SMPL) treatment of diabetic macular edema (DME).

**Methods:** Naïve DME and healthy subjects were enrolled. All DME patients received SMPL treatments (577-nm yellow light, 5% duty cycle of 0.2 s, power 250 mW), according to study protocol. AH of DME eyes was sampled at baseline and periodically after first SMPL treatment. Control eyes were sampled before cataract surgery. Pigment Epithelium Derived Factor (PEDF) and Erythropoietin (EPO) were quantified with glass-chip protein array.

**Results:** Eighteen DME patients (central retinal thickness ≤ 400 μm on Spectral Domain Optical Coherence Tomography (SD-OCT)) and ten controls were enrolled. The main exclusion criteria were high refractive error, proliferative diabetic retinopathy, glaucoma and neurodegenerative disorders. PEDF concentration was decreased in DME patients at baseline versus controls (*P*=0.012), while EPO was increased (*P*=0.029). Both molecules’ concentrations remained stable during follow-up after treatments, compared with DME-baseline.

**Conclusions**: The AH concentrations of RPE biomarkers were significantly different in DME treatment-naïve eyes versus controls. The expression of PEDF and EPO remained unchanged after treatments with SMPL in DME eyes. These data are relevant for future research and applications of SMPL.

## Introduction

Laser photocoagulation has historically represented the major option for the treatment of diabetic macular edema (DME) [1]. Subthreshold Micropulse Laser (SMPL) represents a new tissue-sparing technique [2–4] that prevents the formation of retinal scars, and allows anatomic and functional preservation [2]. It has been hypothesised that SMPL, inducing a finely controlled thermal elevation of the retinal tissue, selectively stimulates the retinal pigment epithelium (RPE) [5–7]. Little is actually known on the precise retinal metabolic changes induced by SMPL. In the last years, proteomic studies on ocular fluids, such as vitreous and aqueous humour (AH) samples, gained greater relevance to study the pathophysiology and the response to treatment of several ocular disorders [8–10]. The samples of AH, which is much more easily accessible than vitreous, has shown promising results in the proteomic approach, not only for anterior segment disorders – where contiguity of AH is high – but also for the posterior pole diseases [8]. It has also been demonstrated that, in simultaneous samples of the same eyes, AH and vitreous proteins concentration are strongly correlated [9,10]. Therefore, AH proteomic analysis is a research procedure that is gaining more interest in ophthalmology, representing a reliable way of detecting specific biomarkers in an easily accessible ocular fluid. Hence, the aim of the present study was to verify the hypothetic effects of SMPL treatment on RPE, using a proteomic analysis of AH samples. Specifically, we evaluated the AH concentrations of two RPE biomarkers, Pigment Epithelium Derived Factor (PEDF) and Erythropoietin (EPO), before and after SMPL treatment in DME eyes.

## Materials and methods

### Study population

Type 2 diabetic patients with previously untreated, non-ischaemic, central involving DME, with central retinal thickness (CRT) ≤ 400 μm (focal or diffuse), were enrolled. The main ocular exclusion criteria were: CRT > 400 μm measured at Spectral Domain Optical Coherence Tomography (SD-OCT); presence of proliferative diabetic retinopathy or significant retinal ischaemia (fluorescein angiography (FA) was performed at baseline and periodically); local treatment or recent (12 months) intraocular surgery; refractive error ≥ 6 dioptres; diagnosis of glaucoma; significant media opacity. The main systemic exclusion criteria were: major neurodegenerative disorders (multiple sclerosis, Alzheimer’s disease etc.); presence of any renal disorders or failure; poor glycaemic and systemic blood pressure control. Healthy subjects, undergoing cataract surgery, served as controls. All patients underwent full ophthalmologic evaluation, best corrected visual acuity (BCVA) evaluation on ETDRS charts, SD-OCT and FA during follow-up visits. The informed consent was obtained for each patient and the research was carried out in accordance with the Declaration of Helsinki regarding experimentation involving human tissue. Local Ethics Committee approval for the study was obtained (protocol number 3194/AO/14).

### Treatment protocol

After pupillary dilation and topical anaesthesia, a single operator applied all laser treatments in all DME eyes, with a Mainster Focal/Grid lens (Ocular Instrument, Bellevue, WA). No titration was performed, as laser setting was the same for all treated eyes, in order to guarantee safety and comparable results [11]. Laser parameters were the same in previous works from our group: 577-nm yellow light (Iridex IQ 577; Laser System Iridex Corp, CA), 5% duty cycle of 0.2 s, power 250 mW [11,12]. SMPL was performed in high-density, fully confluent fashion, over the entire area of retinal thickening at the posterior pole, as usually performed with this laser type [13]. Retreatment was performed 3 months apart, according to study protocol.

### Sample collection, storage and total protein analysis

The AH samples of DME eyes were collected at baseline, and at 1, 3 and 12 months post first SMPL application. Control eyes were sampled once, right before starting cataract surgery (no changes in proteins’ concentration were expected in this group). All patients underwent a standard preoperative procedure. From the anterior chamber, AH (150–200 μl) was aspirated with an insulin syringe (31-gauge needle). The samples were then collected in a vial containing 10 μl of protease inhibitors (Pierce Biotechnology, Rockford, IL, U.S.A.) and stored at −70°C until analysis.

### Protein array

The concentration of PEDF and EPO in the AH samples was measured by means of a customised protein array on glass-chips from RayBiotech™ technology, according to the manufacturer’s instructions (Norcross, GA, U.S.A.). Diabetic and control groups were processed in parallel. Glass slides were processed by GenePix 4400 Microarray scanner (Molecular Devices LLC, Sunnyvale, Silicon Valley, CA, U.S.A.). Fluorescence signals were acquired with the GenePix 4100 microarray scanner (Molecular Devices LLC, Sunnyvale, CA, U.S.A.) equipped with the GenePix pro 3.0 software (Axon Instruments, Foster City, CA, U.S.A.). An inter- and intra-assay coefficient of variability limit of ≤ 10% was set, and a 1.5-fold increase or ≤0.65-fold decrease in signal intensity was considered to guarantee specific signals above background. Fluorescent signals were analysed and fold changes were generated (pathological/control ratio). In order to minimise intra- and inter-assay variability, a single tester handled all the material and followed all experimental phases.

### Statistical analysis

The comparison of AH proteins’ concentration in DME patients at 1, 3 and 12 months and in controls was made, for each protein, by means of Wilcoxon–Mann–Whitney test. Changes in proteins’ expression in DME eyes at 1, 3 and 12 months were separately compared with baseline samples and to controls, and were tested by Wilcoxon’s signed rank sum test. For these two last analyses, Benjamini–Hochberg procedure for multiple-testing correction has been applied choosing a False-Discovery Rate < 20% to accept results as statistically significant. All the analyses have been made using SAS® 9.3 statistical software (SAS-Institute, Cary NC, U.S.A.) on personal computer. *P*-value has been interpreted as statistically significant when <0.05 where not otherwise specified.

## Results

**Figure 1:**
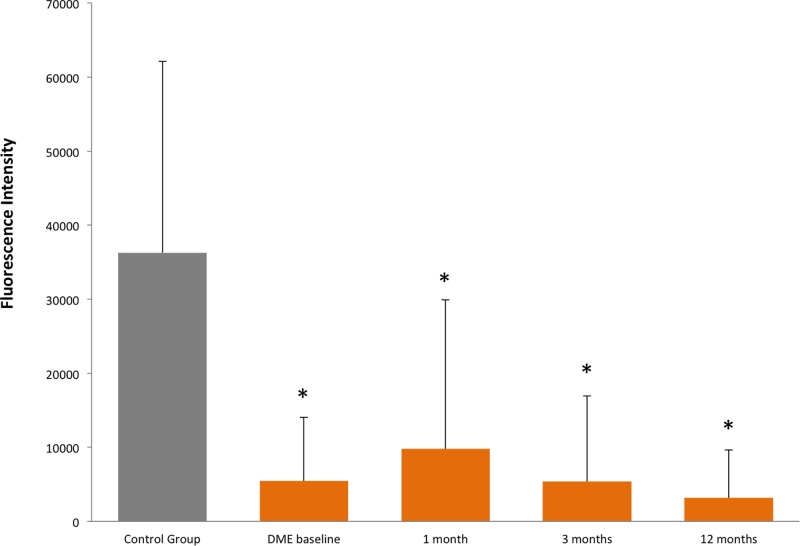
The figure shows AH concentrations of PEDF in healthy subjects and in DME patients at baseline and 1, 3 and 12 months after SMPL treatment The results are expressed as fluorescent intensity signal mean value and standard deviation. The asterisks indicate a significant difference (*P*<0.05) compared to control group (healthy subjects). Note that in DME group no differences were found at 1, 3 and 12 months compared to baseline.

**Figure 2:**
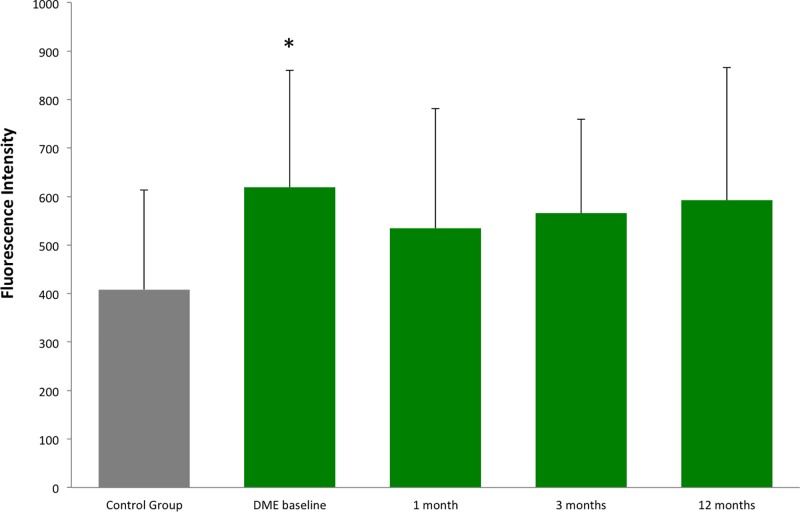
The figure shows AH concentrations of EPO in healthy subjects and in DME patients at baseline and 1, 3 and 12 months after SMPL treatment The results are expressed as fluorescent intensity signal mean value and standard deviation. The asterisk indicates a significant difference (*P*=0.029) compared to control group (healthy subjects). When comparing each time point of DME group after baseline to controls, at 3 months a borderline *P*-value (0.063) was found, while at 1 and 12 months, despite an increased mean value, the significance was not detected. Note that in DME group, no differences were found at 1, 3 and 12 months compared with baseline value.

Eighteen Caucasian diabetic patients with non-proliferative DR and central involving, previously untreated DME were enrolled in the present study. Ten Caucasian, non-diabetic patients, planned for cataract surgery, were also recruited as healthy controls. For each patient one eye was considered in the study analyses. All patients had no concomitant systemic or ocular disorders, and had good blood pressure and glycaemic control. Mean age of DME patients was 63 ± 8.7 years; mean DM duration was 15.2 ± 10.0 years. Mean HbA1c of the three previous months, measured at baseline, was 7.4 ± 2.6%. Mean age of controls was 69 ± 9.8 years. All DME eyes were treated at baseline and at 3, 6 and 9 months with a variable number of spots, depending on DME extension (374.8 ± 168.9 spots per session). At baseline, mean CRT, in the central 1 mm diameter ring, was 360 ± 37.4 µm; mean number of letters on ETDRS score was 77.4 ± 10.1, and significantly increased at 3 and 9 months (+2.9 ± 3.9 and +5.3 ± 8.5 respectively, *P*=0.047). A reduction trend of CRT was also found at the end of study (−23 ± 39.6 µm, *P*=0.078). Total protein content in AH samples remained stable at any moment of analysis. The concentration of PEDF was significantly decreased in DME group at baseline versus controls (*P*=0.012), and remained unchanged during follow-up, without significant concentration changes comparing each time point to DME group at baseline (see Figure 1 and Table 1). The concentration of EPO was significantly increased in DME group at baseline versus controls (*P*=0.029) and no changes after SMPL treatments, compared with baseline, were found (Table 1). At 1, 3 and 12 months, the mean value of EPO’s concentration remained persistently increased versus controls, even if the statistically significant difference was not fully detected (see Figure 2).

**Table 1:** Comparison of PEDF and EPO concentration in DME eyes at 1, 3 and 12 months after SMPL treatment versus baseline

Protein	DME	1 month post	*P*-value	3 months	*P*-value	12 months	*P*-value
	Baseline	SMPL		post SMPL		post SMPL	
**PEDF**	*5466.6 (8589.5)*	*9762.1 (20143.7)*	*0.340*	*5394.4 (11552.1)*	*0.969*	*3164.1 (*6486.9*)*	*0.105*
**EPO**	*619.5 (240.9)*	*534.2 (247.0)*	*0.126*	*565.8 (193.2)*	*0.221*	*592.6 (273.5)*	*0.639*

The results are shown as mean values of fluorescence intensities and standard deviation in brackets. There were no statistically significant differences among all values. *P*-value = Wilcoxon–Mann–Whitney, raw *P*-value and FDR by Benjamini–Hochberg procedure for multiple-testing correction.

## Discussion

In the present study, we applied a proteomic approach on AH samples of DME patients in order to evaluate the effects of SMPL treatment on the concentration of RPE biomarkers after SMPL treatment. Proteomic analysis of ocular fluids, such as vitreous or AH samples, is gaining an increasing interest in ophthalmology, to study both the pathophysiology of still unclear diseases, and the effects of specific treatments, especially intravitreal injections [8]. Even though the major contiguity of vitreous body to the retinal tissue would make vitreous samples hypothetically more reliable to analyse a retinal condition, the dynamics of ocular fluids allow a continuous exchange of solutes between the posterior and anterior compartments [15,16]. For this reason, despite predictable differences of proteins concentration in the AH samples compared to vitreous (that must be taken into account when analysing the proteomic results), both approaches may be considered a reliable way to investigate retinal disorders. Since AH is easily accessible and its sampling is safer and less invasive than vitreous, some research groups are using this approach to study different retinal disorders, as well as to monitor treatment response. For examples, the concentration of specific biomarkers has already been demonstrated increased in AH of diabetic eyes versus healthy subjects [14]. In the present study, we investigated whether PEDF and EPO, biomarkers of RPE activity, undergo any concentration change as a consequence of SMPL application. Surprisingly, we found that the AH concentrations of such RPE markers remained unchanged after successful treatments. In the retinal environment, PEDF is mostly produced by RPE toward the neuroretina, where it exhibits anti-angiogenic and neuroprotective roles [17,18]. In our study, the concentration of PEDF was significantly reduced in DME eyes at baseline compared to controls, confirming many previous reports [17,18]. The concentration of PEDF remained significantly reduced compared to controls at each time point (*P*<0.05 for all measurements). At 1 month follow-up after SMPL treatment, we noticed an increase of the mean value of PEDF concentration, compared to baseline. Despite this increase, a statistically significant difference compared to baseline, was not detected, not only at first control, but also at each time point of follow-up (see Table 1). These data may be due to several factors, such as the low numerosity of the study population, but may also reflect an early reaction of the retinal tissue to SMPL. PEDF is partly produced by other retinal cells, such as Müller cells, which has been recently demonstrated as possible target of SMPL [12]. Since, however, a statistically significant change of PEDF concentration compared to baseline was never detected, the main production of PEDF (from RPE) does not seem influenced by SMPL treatments. We also evaluated the concentration of EPO in the same eyes. Although few studies investigated retinal EPO expression, some authors agree that EPO represents a specific biomarker of RPE activity [19–22]. Our study found an increased value of EPO in naïve DME patients versus controls, confirming previous reports [20]. After treatments, no changes in EPO’s concentration were detected in the AH samples compared with baseline value. Even though at 1 month follow-up, a mild decrease in mean value of EPO was found compared to baseline, however, no statistically significant difference was reached, comparing each time point of follow-up to baseline value (Table 1). A similar consideration, as for PEDF, is that the early (1 month) change of EPO concentration may be due to a mild response of retinal tissue, with a slight movement of proteins concentration that, however, was not statistically significant.

Our results seem to confirm the efficacy of SMPL treatment in DME, as shown by the reduction trend of CRT and the significant improvement of BCVA. However, proteomic results showed that AH levels of two specific RPE biomarkers were not significantly influenced by SMPL treatments in our study population. To our knowledge, this is the first study on RPE biomarkers changes after SMPL in a human model. These data may significantly contribute to elucidate the SMPL mechanism of action, with possible implications for future treatment applications. Larger studies would be desirable to confirm and deepen these preliminary results.

## Abbreviations

AH: aqueous humour
BCVA: best corrected visual acuity
CRT: central retinal thickness
DME: diabetic macular edema
DR: Diabetic Retinopathy
EPO: erythropoietin
ETDRC: Early Treatment Diabetic Retinopathy Study
FA: fluorescein angiography
FDR: false discovery rate
PEDF: pigment epithelium derived factor
RPE: retinal pigment epithelium
SD-OCT: spectral domain optical coherence tomography
SMPL: subthreshold micropulse laser
